# Dropsey from Malaria

**Published:** 1881-02

**Authors:** W. H. Jenney

**Affiliations:** Iowa City; Kansas City, Mo.


### DROPSY FROM MALARIA.

*A Case in Practice.*

BY W. H. JENNEY, M.D., KANSAS CITY, MO.

Mr. P——, the subject of this article, came under my care, from allopathic hands, July 15th, 1880, and has been treated by me since that date. I will first give his statement direct:

"In October 1877, I was affected with malaria and chills, which ended in neuralgia of the head (mostly about the vertex). Was treated by an allopathic physician who gave me, to start with, forty grains of quinine in two hours' time, which occasioned great distress and nausea; this was allowed to act until its effects had passed off—about twenty-four hours. I was then obliged to take thirty grains a day for one week, with no abatement of the neuralgia, but with high and constant fever. After a rest of one week the quinine was resumed; but I grew no better, which resulted in confining me to the house for four months, without alleviation from pain except when under the influence of morphine. Had a consultation of physicians, who determined I had but a slight prospect of recovery. At the expiration of five months, and after taking more than six hundred grains of quinine (with morphine, etc., as usually prescribed by the allopaths), I began to improve, so that by the sixth month I was able to resume my duties as an expressman, remaining in charge of my office until October 1878, having been at work since May, 1878. I had attacks of neuralgia until September 1879, when my feet commenced swelling, extending to the limbs and body and having the appearance of dropsy. Consulted my physician again, calling attention to said dropsy. He called it weakness, and predicted it would disappear as soon as my strength returned; the swelling, however, continued until my weight increased from one hundred and twenty-five to one hundred and seventy pounds. For this condition I took large doses of elaterium, which so deranged my stomach I had to discontinue it. After this I was confined to my bed for two weeks, being told that all now would be right, and being urged to continue the elaterium, which I could not do, on account of nausea. I now changed physicians; and was given, among other things, large doses of jaborandi, but continued to fill up at the rate of two to five pounds per day, my weight increasing to one hundred and eighty pounds. On October 16th, 1879, I was tapped, twenty pints of water being removed; in fifteen days was again tapped, but continued to fill again. My physician again resorted to elaterium, with the same results—nausea and debility. Was again tapped and relieved of twenty-two pints of fluid. On November 15th I refused to take any more medicine, and was given up to die, to which I did not object. Took a mild tonic after this, and by the last of December was able to sit up for a time, finally becoming able to walk. The tapping was continued, however, until I had been operated upon thirty times, and parting with five hundred and ninety-six and a half pints of water!"

The history of this case is certainly interesting, and bears a moral—that however desperate a case may be not to give up so long as we possess our wonderful Materia Medica, and are able to find the *similimum* remedy. When called to this case, it was with great hesitancy that I took charge of it; the patient seeming to have but little chance for recovery. The dropsical effusion extended from the feet to the chest, causing much distress from distension of the bowels and impingement on the diaphragm. On being called the period for tapping had arrived, and I endeavored to extend the time, succeeding by means of *Arsenicum*, in holding my case, but had to resort to tapping later, drawing off twenty-one and a half pounds of fluid. But I continued with *Arsenicum*, there being constant and intense thirst, with fear of drinking, lest there should be faster accumulation of dropsical fluid. I continued this remedy until the end of three weeks, and tapped again, this time removing twentythree and a half pounds of water. Examined the dropsical fluid, and found a heavy precipitate of albumen; after standing over night, found a column of solid albumen the size of a cigar and two inches high. Studying now my case closely, I determined that *Apis* was *the* remedy, the thirst arising from dryness of the fauces; he had great restlessness and despaired of getting well; urine was diminished and high colored, swollen extremities, etc. The patient continued to swell, as before, until the third week, when he desired to be relieved by tapping. Now re-examined my patient and concluded to continue *Apis*, it being apparent that the effusion in the limbs was decreasing. From this time the patient continued to improve, losing bulk and weight until the eighth week, when the flesh began to increase and solidify. Now, after four months, he appears well, weighing one hundred and forty-five pounds, and has resumed his business, a sound man, physically and mentally.

